# Determining Rheumatology Patient Interest in a Group Strength Training Program - Results of an Exercise Survey

**DOI:** 10.23937/2469-5718/1510121

**Published:** 2019-02-20

**Authors:** Kevin Rhie, Danielle M Feger, Rayford R June, Christopher N Sciamanna, Sharon E Banks

**Affiliations:** Department of Internal Medicine, Penn State Health, USA

**Keywords:** Group, Strength, Rheumatology, Exercise, Interest

## Abstract

**Background:**

Exercise has proven benefits in rheumatologic disease including reducing inflammation and improving symptoms. A Group Strength Training (GST) program design has improved adherence to exercise in primary care patients but the effect is unknown in rheumatology patients. We examined the interest of rheumatology patients with different diagnoses and the effect of comorbidities in pursuing an organized GST program.

**Methods:**

We conducted a cross-sectional survey of patients from a rheumatology practice in central Pennsylvania in February and April 2017. This survey assessed self-reported interest of patients in a GST program in addition to demographics, comorbidities, and quality of life measures. Primary care data from a previous survey were used for comparative analysis for the primary outcome: interest in a GST program.

**Results:**

Fifty percent of rheumatology patients were interested in a GST program and there was no difference of interest compared to primary care patients (X^2^ = 2.04, *p* = 0.15). There was no difference in interest in a GST program for rheumatology patients with poor health compared to patients with good health (OR = 0.9, *p* = 0.8). Female patients were more interested in a group strength training program than male patients (OR = 3.7, *p* = 0.001). Patients with a BMI of 25–30 (OR = 2.2, *p* = 0.04) or > 30 (OR = 1.7, *p* = 0.12) were more interested compared to those with a normal BMI. There was no difference in interest in group strength training regardless of rheumatology diagnosis or comorbidities.

**Conclusion:**

Our data suggest that rheumatology patients are interested in a GST program regardless of disease, medical comorbidities, perceived mental or physical health, or education level. Further study is needed to determine the effects of GST on rheumatologic diseases.

## Introduction

Patients treated in rheumatology practices suffer from a wide variety of debilitating diseases including but not limited to Rheumatoid Arthritis (RA), Systemic Lupus Erythematosus (SLE), Psoriatic Arthritis (PsA), Ankylosing Spondylitis (AS), and Sjogren’s Syndrome (SS). Medical treatment of these diseases typically targets the inflammatory pathway with the progressive use of immunosuppressant medications [1,2]. These medications, while effective, are also associated with complications such as opportunistic infections and medication related side effects [1,3]. Adherence and availability of medications are limited secondary to high costs especially of biologic medications [3,4].

The benefit of exercise as a treatment modality is not a novel concept. It is known that exercise can decrease inflammation in both healthy patients and patients with rheumatic disease [5]. It can also decrease disease activity, fatigue, pain; improve strength, function, and have a positive effect on rheumatology patients’ overall health and quality of life [5–10]. Resistance training in particular has been shown in rheumatology patients to be associated with improvement in aerobic capacity and muscle strength [6,11]. In addition, rheumatology patients can have an increased incidence of cardiovascular disease and poor physical fitness is a predictor of increased mortality in rheumatology patients [12,13]. However, patient engagement in physical activity through exercise programs remains a problem in rheumatology clinics. Rheumatology patients often do not exercise regularly [14] and patients often avoid physical activity secondary to misinformation and fear [15]. The feasibility or benefit of a strength training program in rheumatology patients has not been previously well described, and strength training specifically is generally not suggested as a specific treatment recommendation perhaps due to lack of evidence. A rheumatology patient specific community Group Strength Training (GST) program may provide a social atmosphere to improve adherence to exercise which can then provide an adjunctive therapy for rheumatology patients in addition to medications to help control disease activity. It is unknown if a GST program involving only rheumatology patients would improve adherence or potentially offer a non-pharmacologic treatment intervention for patients with rheumatic disease. We conducted a survey to evaluate patient interest in a GST program. Our primary objective was to investigate rheumatology patient interest in a rheumatology patient specific GST program intervention. We hypothesized that rheumatology patients regardless of disease would be interested in GST programs.

## Methods

A validated health and behaviors survey used in primary care was modified for a rheumatology specific population within the same health system [16] (Appendix 1). The questionnaire included questions to assess patient interest in a GST program, demographic information, and self-reported quality of life measures. The anonymous survey was approved as a quality improvement project without formal review by the Penn State College of Medicine institutional review board. All patients at two rheumatology clinics in central Pennsylvania were provided surveys during the months of February and April 2017.

Interest in a GST program was assessed using the question, “Would you consider participating in any of the following: A group program to increase your muscle strength” with “Yes” or “No” responses. Mental and physical quality of life were assessed using the following questions from the Healthy Days Measure from the Centers for Disease Control and Prevention, “Thinking about your physical health, which includes physical illness and injury, for how many days during the past 30 days was your physical health NOT good? Now thinking about your mental health, which includes stress, depression, and problems with emotions, for how many days during the past 30 days was your mental health NOT good?” [17] Self-reported health was assessed using the question which has been shown to predict future hospitalization and death, “In general, how is your health”, with available responses including: “Excellent”, “Very Good”, “Good”, “Fair”, and “Poor” [18]. Physical activity was assessed using the questions developed by Greenwood, et al. and the National Institute of Health Interview Survey, “How many days during the past week did you perform physical activities where your heart beats faster and your breathing is harder than normal for 30 minutes or more? How many days during the past week did you perform physical activities specifically designed to strengthen your muscles such as lifting weights or doing calisthenics”, to measure both aerobic and anaerobic activity respectively [19,20]. Choices for diagnoses included rheumatoid arthritis, systemic lupus erythematosus, psoriatic arthritis, osteoarthritis, fibromyalgia, ankylosing spondylitis, Sjogren’s syndrome and “other”. Respondents were able to indicate multiple diagnoses. Co-morbidity choices were limited to hypertension, diabetes, hyperlipidemia, and coronary artery disease; subjects were asked to report “Yes” or “No” for each comorbid diagnosis. All demographics and disease characteristics were self-reported.

Chi-square tests of homogeneity were used to test for differences between categorical variables. A multivariable logistic regression model was used to quantify interest in a GST, including all variables of interest regardless of statistical significance at the univariate level. All statistical tests were two-sided with a significance level of 0.05, and all analyses were performed in SAS 9.4 (Cary, NC). Responses were excluded from analyses if surveys were not returned or if they were returned but did not include enough responses for the statistical model.

## Results

A total of 397 of 656 patients returned surveys with a response rate of 61.6%. Patients had a mean age of 52 and 80% were female (Table 1). The most common rheumatic diagnoses were RA (39.0%), fibromyalgia (FMS) (21.4%), and Osteoarthritis (OA) (19.7%). Twenty-nine percent of subjects had a Body Mass Index (BMI) ≤ 25, 28% had a BMI of 25–30, and 44% had a BMI > 30. Over one third of patients reported high cholesterol (33.5%) or hypertension (40.3%) as medical co-morbidities.

The average reported number of days of aerobic exercise over the previous week was 2.2. The average reported number of days of strength training was 1.4 with the majority of patients (58.8%) reporting 0 days. A majority of patients (72.7%) reported at least one day of aerobic or strength exercise over the previous week.

Only 3% of patients rated their overall health as excellent, while 19% rated their health as very good, 35% as fair, and 11% as poor. Most (62.2%) patients reported at least 10 days of poor physical health per month and 44.5% of patients reported at least 10 days of poor mental health per month.

Approximately half (50.1%) of rheumatology patients were interested in a GST program. Female rheumatology patients were more interested in GST than male patients (Odds Ratio [OR] = 3.7, *p* = 0.001). A lower percentage of men (31%) versus women (55%) were interested in GST. Patients with a BMI of 25–30 (OR = 2.2, *p* = 0.04) or > 30 (OR = 1.7, *p* = 0.12) were also more interested compared to those with a normal BMI. Between those interested in GST and those not, there was no difference in average age (51.6 years vs. 51.1 years, *p* = 0.95), proportion of Caucasians (85% vs. 87%, *p* = 0.55), proportion of Hispanics (8% vs. 9%, *p* = 0.63), presence of comorbidities, aerobic exercise days over the previous week (2.3 vs. 2.0 days, p = 0.63), strength exercise days over the previous week (1.4 days vs. 1.4 days, p = 1.00), days of poor physical health per month (13.7 days vs. 13.0 days, p = 0.96), and days of poor mental health per month (9.8 days vs. 9.4 days, *p* = 0.99). There was no difference in interest in a GST for rheumatology patients with “Fair” or “Poor” health compared to patients with “Excellent”, “Very Good”, or “Good” health (OR = 0.9, *p* = 0.8, Table 2). Notably, there was also no difference in GST interest between rheumatic diagnoses (Table 3).

## Discussion

It is known that rheumatology patients exercise less than their healthy counterparts and previous studies have estimated that 80% of rheumatology patients are physically inactive [12,14]. Barriers to physical activity include physical limitations such as pain, fatigue, and physical capability as well as psychological aspects such as lack of enjoyment, motivation, confidence, and fear [15,21]. In our survey, when compared to a similar survey by Sciamanna, et al. [15] of general internal medicine patients, there was no difference with an unadjusted chi-square test in the number of rheumatology patients interested in GST (55%, *p* = 0.15). Interestingly, perceived “Fair” or “Poor” health was higher in rheumatology patients (45.1%) compared to GIM patients (18.8%, *p* < 0.0001) implying that rheumatology patients have worse perceived general health than published GIM patients [16]. This is consistent with previous studies that have shown a substantial impact of rheumatologic disease on physical and mental health [22]. But despite these physical barriers to engaging in physical activity, a majority of rheumatology patients (50.1%) are nonetheless still interested in GST. We believe this to be because of several reasons. First, a rheumatology specific program may be more beneficial than a typical exercise program designed for healthy patients and this may lead to improved adherence. A previous study has shown that a tailor-made exercise program for patients with knee OA had an adherence rate of greater than 90% [23]. Rheumatology patients may typically avoid traditional exercise programs which do not consider the disease specific physical limitations of these patients [21]. In addition, exercising in a group can provide a social environment and encourage patients to be more engaged in physical activity. This social aspect is likely what makes group activities such as spin classes, yoga classes, dance fitness, and SilverSneakers® popular. In fact, exercising in group settings has been shown to have more health benefits compared to exercising alone [24]. It is not surprising that certain group fitness classes are popular among females and in our findings, females were more likely to be interested GST. In addition, people with physical limitations related to their rheumatologic disease would be supportive of others in a group who would have similar limitations.

Common cardiovascular risk factors such as diabetes, hypertension, hyperlipidemia, and coronary artery disease were unsurprisingly also common in our surveyed rheumatology patients which are consistent with findings from a previous study [25]. It is well known that exercise can help treat these conditions [26]. Overweight patients (BMI 25–30) may be interested to adopt healthy lifestyle choices for additional reasons outside of their rheumatologic disease and have the most to gain from exercise.

This survey does have limitations. Data were only collected in central Pennsylvania and results may not be generalizable to other geographic settings. There is a possibility of response bias as respondents self-reported their diagnosis. In addition, within a rheumatology practice, there is a wide range of rheumatology diagnoses and varying degrees of disease activity which can affect the interest level of patients. Objectively measuring disease activity was not possible with this survey to assess if higher or lower disease activity is associated with being interested in a GST program. However, we do know that overall health was fair/poor in our surveyed patients which is similar to other studies which showed rheumatology patients have overall poor health related quality of life [22]. The rates of fair/poor health are quite high in this clinic compared to primary care practices. In theory, a GST program would only be offered to patients with stable disease and physically able to participate since these are the patients who would be more likely to adhere to an exercise program. A respondent’s prior experience with GST or any other exercise program may have impacted his or her current interest in a GST program depending on whether this prior experience was positive or negative. This survey did not assess for respondents’ prior experience with GST and this is an area for future investigation to further determine barriers to participating in GST and improve adherence.

Even though the majority of questionnaire respondents showed an interest in a GST program, we recognize that it is only a slim majority and there are still many patients who are not interested. Finding methods to engage these patients who are not interested and increasing interest in GST program is a foreseeable challenge. Even for the patients who are interested, we hope that interest will translate into future participation but there are still obstacles to overcome. Logistical challenges such as a convenient location and time will certainly affect an individual’s ability to participate in a GST program.

Despite these limitations, our findings suggest that rheumatology patients may be interested in GST regardless of disease, age, medical comorbidities, perceived mental or physical health or education level. A particular emphasis in program development may be placed on female and overweight patients as they showed an increased interest in GST programs. Future studies should examine if a GST program design improves exercise adherence in rheumatology patients.

## Conclusions

This study shows that rheumatology patients are interested in GST across a wide range of diagnoses, comorbidities, and perceived general health. It appears that women in rheumatology clinics are more interested in a GST program. Interest in a GST program does not necessarily lead to better adherence and future studies should investigate the barriers to participation and methods to increase adherence. Designing an exercise programing specifically for rheumatology patients needs special care. Future studies are needed to determine the effects of GST on disease activity in rheumatic disease and the overall health of rheumatology patients.

## Supplementary Material

Appendix 1

## Figures and Tables

**Table 1:** Demographics.

Item	Overall N = (%)	Rheumatoid Arthritis N = (%)	Osteoarthritis N = (%)	Fibromyalgia N = (%)
*Age (Years)*	52.0 (SD = 15.4)	53.2 (SD = 15.0)	60.0 (SD = 11.5)	48.0 (SD = 13.5)
18–44	121 (32.4%)	39 (26.9%)	6 (8.0%)	31 (38.3%)
45–54	81 (21.7%)	36 (24.8%)	18 (24.0%)	25 (30.9%)
55–64	91 (24.3%)	28 (26.2%)	22 (29.3%)	18 (22.2%)
≥ 65	81 (21.7%)	32 (22.1%)	29 (38.7%)	7 (8.6%)
Female	300 (80.0%)	113 (77.9%)	64 (85.3%)	73 (90.1%)
Caucasian	323 (81.2%)	121 (78.1%)	66 (84.6%)	72 (84.7%)
Hispanic	34 (9.2%)	14 (9.7%)	6 (8.1%)	8 (10.1%)
Current Smoker	66 (17.2%)	25 (17.0%)	11 (14.3%)	21 (26.3%)
*Level of Education*				
< High School	24 (6.5%)	14 (9.8%)	6 (8.0%)	4 (4.9%)
High School	115 (31.2%)	42 (29.4%)	22 (29.3%)	23 (28.1%)
Some College	116 (31.4%)	47 (32.9%)	30 (40.0%)	40 (48.8%)
College or Higher	115 (31.1%)	40 (28.0%)	17 (22.7%)	15 (18.3%)
BMI (kg/m^2^)	30.2 (SD = 7.9)	29.9 (SD = 7.9)	32.0 (SD = 9.1)	31.2 (SD = 7.8)
≤ 25	100 (28.6%)	39 (29.1%)	12 (17.9%)	16 (21.9%)
25–30	97 (27.7%)	41 (30.6%)	22 (32.8%)	19 (26.0%)
> 30	153 (43.7%)	54 (40.3%)	33 (49.3%)	38 (52.1%)
*Comorbidities*				
Diabetes	52 (14.2%)	20 (14.5%)	13 (17.8%)	10 (13.0%)
High Cholesterol	123 (33.5%)	46 (32.9%)	34 (47.2%)	22 (28.2%)
Hypertension	151 (40.3%)	53 (37.1%)	38 (52.1%)	31 (38.8%)
Coronary Artery Disease	34 (9.2%)	14 (9.7%)	8 (11.0%)	5 (6.5%)
Fibromyalgia	85 (21.4%)	26 (16.8%)	31 (41.0%)	85 (100.0%)
*Days of Aerobic Exercise/Week*	2.2 (SD = 2.3)	2.0 (SD = 2.2)	2.0 (SD = 2.1)	2.3 (SD = 2.3)
0	132 (36.8%)	57 (40.7%)	24 (35.3%)	26 (34.7%)
1–3	133 (37.1%)	50 (35.7%)	30 (44.1%)	28 (37.3%)
4–7	94 (26.2%)	33 (23.6%)	14 (20.6%)	21 (28.0%)
*Days of Strength Exercise/Week*	1.4 (2.0)	1.5 (2.1)	1.1 (1.7)	1.0 (1.7)
0	211 (58.8%)	78 (56.9%)	44 (61.1%)	51 (64.6%)
1–3	94 (26.2%)	37 (27.0%)	20 (27.8%)	19 (24.1%)
4–7	54 (15.0%)	22 (16.1%)	8 (11.1%)	9 (11.4%)
*Physical Activity*				
None	99 (27.3%)	44 (31.4%)	18 (25.0%)	23 (29.5%)
1+ Days/Week	264 (72.7%)	96 (68.6%)	54 (75.0%)	55 (70.5%)
*General Health Rating*				
Excellent	11 (2.8%)	2 (1.3%)	1 (1.3%)	0 (0.0%)
Very Good	75 (19.2%)	23 (15.1%)	8 (10.3%)	6 (7.2%)
Good	128 (32.8%)	51 (33.6%)	32 (41.0%)	23 (27.7%)
Fair	135 (34.6%)	57 (37.5%)	26 (33.3%)	36 (43.4%)
Poor	41 (10.5%)	19 (12.5%)	11 (14.1%)	18 (21.7%)
*Days of Poor Physical Health/Month*	13.6 (SD = 10.6)	14.2 (SD = 10.4)	13.7 (SD = 10.7)	18.0 (SD = 9.6)
0	49 (13.9%)	17 (12.6%)	8 (12.5%)	4 (5.3%)
1–9	84 (23.9%)	28 (20.7%)	17 (26.6%)	11 (14.7%)
≥ 10	219 (62.2%)	90 (66.7%)	39 (60.9%)	60 (80.0%)
*Days of Poor Mental Health/Month*	9.8 (SD = 10.7)	9.2 (SD = 10.6)	9.7 (SD = 10.6)	13.6 (SD = 11.3)
0	117 (33.0%)	54 (39.7%)	24 (35.8%)	12 (15.6%)
1–9	80 (22.5%)	24 (17.7%)	13 (19.4%)	20 (26.0%)
≥ 10	158 (44.5%)	58 (42.7%)	30 (44.8%)	45 (58.4%)

**Table 2:** Comparison between persons interested in Group Strength Training and not interested.

Item	Interest N = (%)	No Interest N = (%)	P-value
Age (Years)	51.6 (SD = 14.8)	51.5 (SD = 16.3)	0.95
18–44	57 (48.7%)	60 (51.3%)	
45–54	36 (46.2%)	42 (53.9%)	
55–64	47 (57.3%)	35 (42.7%)	
≥ 65	35 (48.0%)	38 (52.1%)	
Female	155 (88.1%)	127 (73.0%)	0.0004^†^
Caucasian	150 (85.2%)	153 (87.4%)	0.55
Hispanic	14 (8.0%)	16 (9.4%)	0.63
Current Smoker	26 (44.1%)	33 (55.9%)	0.28
*Level of Education*			0.11
< High School	6 (28.6%)	15 (71.4%)	
High School	49 (45.8%)	58 (54.2%)	
Some College	61 (55.5%)	49 (44.6%)	
College or Higher	56 (51.9%)	52 (41.2%)	
BMI (kg/m^2^)	31.1 (SD = 7.9)	29.7 (SD = 8.1)	0.10
≤ 25	39 (42.9%)	52 (57.1%)	
25–30	46 (51.7%)	43 (48.3%)	
>30	84 (56.0%)	66 (44.0%)	
*Comorbidities*			
Diabetes	25 (56.8%)	19 (43.2%)	0.44
High Cholesterol	66 (56.4%)	51 (43.6%)	0.17
Hypertension	72 (53.7%)	62 (46.3%)	0.42
Coronary Artery Disease	13 (40.6%)	19 (59.4%)	0.22
Fibromyalgia	38 (48.7%)	40 (51.3%)	0.78
*Days of Aerobic Exercise/Week*	2.3 (SD = 2.2)	2.0 (SD = 2.3)	0.63
0 (%)	55 (46.6%)	63 (53.4%)	
1–3 (%)	66 (51.6%)	62 (48.4%)	
4–7 (%)	47 (54.7%)	39 (45.4%)	
*Days of Strength Exercise/Week*	1.4 (SD = 2.0)	1.4 (SD = 2.1)	1.00
0 (%)	94 (48.7%)	99 (51.3%)	
1–3 (%)	51 (56.0%)	40 (44.0%)	
4–7 (%)	22 (44.9%)	27 (55.1%)	
*Physical Activity*			0.28
None	42 (46.2%)	49 (53.9%)	
1+ Days/Week	130 (52.9%)	116 (47.2%)	
*General Health Rating*			0.16
Excellent (%)	2 (20.0%)	8 (80.0%)	
Very Good (%)	30 (45.5%)	36 (55.6%)	
Good (%)	63 (53.9%)	54 (46.2%)	
Fair (%)	64 (53.8%)	55 (46.2%)	
Poor (%)	13 (40.6%)	19 (59.4%)	
*Days of Poor Physical Health/Month*	13.7 (SD = 10.0)	13.0 (SD = 10.8)	0.96
0 (%)	18 (40.9%)	26 (59.1%)	
1–9 (%)	39 (49.4%)	40 (50.6%)	
≥ 10 (%)	108 (54.6%)	90 (45.5%)	
*Days of Poor Mental Health/Month*	9.8 (10.6)	9.4 (10.7)	0.99
0 (%)	51 (47.7%)	56 (52.3%)	
1–9 (%)	43 (55.8%)	34 (44.2%)	
≥ 10 (%)	74 (52.1%)	68 (47.9%)	

† *P* < 0.05.

**Table 3:** Comparison between persons interested in Group Strength Training and not interested by diagnosis.

Item	Interested N = (%)	Not Interested N = (%)	P-value
Rheumatoid Arthritis	64 (48.9%)	67 (51.2%)	0.71
Lupus	28 (58.3%)	20 (41.7%)	0.22
Psoriatic Arthritis	16 (48.5%)	17 (51.5%)	0.84
Osteoarthritis	34 (50.0%)	34 (50.0%)	0.98
Fibromyalgia	38 (48.7%)	40 (51.3%)	0.78
Ankylosing Spondylitis	8 (57.1%)	6 (42.3%)	0.59
Sjogren’s Syndrome	17 (54.8%)	14 (45.2%)	0.58

## List of Abbreviations

RA: Rheumatoid Arthritis
SLE: Systemic Lupus Erythematosus
PsA: Psoriatic Arthritis
AS: Ankylosing Spondylitis
SS: Sjogren’s Syndrome
GST: Group Strength Training
OA: Osteoarthritis
FMS: Fibromyalgia
BMI: Body Mass Index
GIM: General Internal Medicine
