# Supplementary material for: Determining Rheumatology Patient Interest in a Group Strength Training Program - Results of an Exercise Survey
Source: Int J Sports Exerc Med. Author manuscript; Available in PMC 2019 Jul 19. (PMC6641551; doi:10.23937/2469-5718/1510121)
Supplement: Appendix 1 [file NIHMS1019014-supplement-Appendix_1.pdf]

PennState Health
Milton S. Hershey Medical Center

This survey will help us know what programs to offer to keep our patients healthy. Your answers are ANONYMOUS. Thank you very much!

What Diagnosis/ Diagnoses are you being seen for today (check all that apply) ?

__ Rheumatoid Arthritis ___ Lupus __ Psoriatic Arthritis ___ Osteoarthritis __ Fibromyalgia ___Ankylosing Spondylitis ___ Sjogrens Syndrome __ Other

In general, how's your health? ___ excellent | ___ very good | ___ good | ___ fair | ___ poor

Thinking about your physical health, which includes physical illness and injury, for how many days during the past 30 days was your physical health NOT good? ______ NUMBER OF DAYS FROM 0 to 30

Now thinking about your mental health, which includes stress, depression, and problems with emotions, for how many days during the past 30 days was your mental health NOT good? ______ DAYS FROM 0 to 30

By yourself, and without using any special equipment, how difficult is it for you to…

	**Not difficult at all**	**Only a little difficult**	**Somewhat difficult**	**Very difficult**	**Can't do it at all**
Walk a quarter of a mile – about 3 city blocks?	1	2	3	4	5
Lift or carry something as heavy as 10 pounds?	1	2	3	4	5

How many days during THE PAST WEEK did you perform physical activities where your heart beats faster and your breathing is harder than normal for 30 minutes or more? ___ NUMBER OF DAYS FROM 0 to 7

How many days during THE PAST WEEK did you perform physical activities specifically designed to strengthen your muscles such as lifting weights or doing calisthenics? ___ NUMBER OF DAYS FROM 0 to 7

Do you smoke cigarettes every day? ___ yes | ___ no?

Have you ever been told by a doctor that you had?

Diabetes?	___ YES	___ NO
High cholesterol?	___ YES	___ NO
Hypertension (high blood pressure)?	___ YES	___ NO
A heart attack or coronary artery disease?	___ YES	___ NO

PLEASE TURN THIS OVER TO ANSWER A FEW MORE QUESTIONS

These questions will help us understand what programs to offer to our patients and advocate for.

Would you consider participating in any of the following?

Doing a short (5 km or less) walk or run regularly with others who had had arthritis?	___ YES	___ NO
A group program to increase your muscle strength?	___ YES	___ NO
A regular fitness program where people your own age played games, that were made to be easier to play and less competitive?	___ YES	___ NO
A focus group that can brainstorm about new ideas to increase fitness in arthritis patients ?	___ YES	___ NO
Learning about and following a diet to improve your health?	___ YES	___ NO

What is your age? __________ years

How tall are you without shoes? ______ feet | _______ inches

How much do you weigh without clothes or shoes? _______ pounds

What is your gender? ___ female | ___ male

Are you Hispanic or Latino? ___ yes | ___ no

Do you do any paid work? ___ yes | ___ no

Which one of the following best describes your race?

____ White	____ Black or African-American	____ Asian, Hawaiian or Pacific Islander	____ Other

What is the highest grade or year of school you completed?

____ Did not graduate high school	____ Grade 12 or GED (High school graduate)	____ College 1 year to 3 years (some college)	____ College 4 years or more (college graduate)

WHEN FINISHED, PLEASE FOLD AND PLACE IN THE SURVEY BOX. THANK YOU!!
